# In Vitro Antibacterial Activity of Several Plant Extracts and Oils against Some Gram-Negative Bacteria

**Published:** 2014-01

**Authors:** Ayman Al-Mariri, Mazen Safi

**Affiliations:** 1 Department of Molecular Biology and Biotechnology, Atomic Energy Commission, Damascus Syria

**Keywords:** Gram-negative bacteria, Antibiotic resistance, *Cinnamomum zeylanicum*, *Syzygium aromaticum*

## Abstract

**Background: **Medicinal plants are considered new resources for producing agents that could act as alternatives to antibiotics in the treatment of antibiotic-resistant bacteria. The aim of this study was to evaluate the antibacterial activity of 28 plant extracts and oils against four Gram-negative bacterial species.

**Methods: **Experimental, in vitro*,* evaluation of the activities of 28 plant extracts and oils as well as some antibiotics against *E. coli* O157:H7, *Yersinia* *enterocolitica* O9, *Proteus* spp*.*, and *Klebsiella* *pneumoniae* was performed. The activity against 15 isolates of each bacterium was determined by disc diffusion method at a concentration of 5%. Microdilution susceptibility assay was used in order to determine the minimal inhibitory concentrations (MICs) of the plant extracts, oils, and antibiotics.

**Results: **Among the evaluated herbs, only *Origanum syriacum *L*.*,* Thymus syriacus* Boiss.,* Syzygium aromaticum* L.,* Juniperus foetidissima Wild*, *Allium sativum *L., *Myristica fragrans *Houtt, and* Cinnamomum zeylanicum *L*. *essential oils and *Laurus nobilis* L. plant extract showed anti-bacterial activity. The MIC_50_ values of these products against the Gram-negative organisms varied from 1.5 (*Proteus *spp. and* K. pneumoniae*( and 6.25 µl/ml (*Yersinia* *enterocolitica* O9 ) to 12.5 µl/ml (*E. coli *O:157).

**Conclusion: **Among the studied essential oils, *O. syriacum *L.,* T. syriacus *Boiss.,* C. zeylanicum *L., and *S. aromaticum* L. essential oils were the most effective. Moreover, Cephalosporin and Ciprofloxacin were the most effective antibiotics against almost all the studied bacteria. Therefore, *O. syriacum *L.,* T. syriacus *Boiss.,* C. zeylanicum *L., and *S. aromaticum* L. could act as bactericidal agents against Gram-negative bacteria.

## Introduction

Medicinal and aromatic plants are used on a large scale in medicine against drug-resistant bacteria, which are considered one of the most important reasons for the lack of success of treatment in infectious diseases. Medicinal plants are the major sources of new medicines and may constitute an alternative to the usual drugs.^1^

Aromatic oils are used in many industries, including food preservation,^2^ pharmacy, and medicine.^3^^,^^4^ They are expected to form new sources of antimicrobial drugs, especially against bacteria.^5^ The antibacterial effectiveness of aromatic oils has been divided into a good, medium, or bad.^6^^,^^7^ These oils can also produce some defense products against several natural enemies.^8^ In addition, and in order to continue their natural growth and development, aromatic oils may produce some secondary metabolites in response to some external stress.^9^

The extracts and oils of 28 plants used in this work have been traditionally employed by people for various purposes in different parts of the world.* Cinnamomum zeylanicum* essential oil has antibacterial and antifungal activities^10^ as well as anti-diabetic properties;^11^ *Citrus limon* and *Rosmarinus officinalis *L*. *essential oils possess antioxidant properties;^12^^,^^13^ *Citrus aurantium* has immunological effects in humans;^14^ *Eucalyptus globulus* oil has good antimicrobial activities;^15^^,^^16^ *Thymus pannonicus* essential oil has an excellent effect against *E. coli* O157:H7;^17^ light thyme essential oil inhibits the growth of *E. coli* O157:H7 in foods;^18^ *Brillantaisia lamium* extract exhibits antibacterial and antifungal effects against *Staphylococcus aureus, Enterococcus faecalis, Candida tropicalis*, and *Cryptococcus neoformans*;^19^ and finally *Crinum purpurascens *herb extract has antimicrobial activities against *Salmonella paratyphi *A and B.^20^ Traditionally, many plant extracts and oils are used as medicinal plants in Syria for many purposes, particularly for respiratory and gastrointestinal disorders.

The aim of this study was to screen the in vitro antibacterial activity of 28 plant extracts and oils against some Gram-negative bacteria, including: *E. coli* O157:H7, *Yersinia* *enterocolitica* O9, *Proteus* spp*.*, and *Klebsiella* *pneumoniae*.

## Materials and Methods

*Microorganisms and Growth Conditions* **

Fifteen local isolates of *E. coli *O157:H7,* Y. enterocolitica *O9,* Proteus *spp., and* K. pneumoniae* were grown for 24-48 h in 2YT agar (peptone, 16 g/liter; yeast extract, 10 g/liter; NaCl, 5 g/liter; agar, 13 g/liter [Difco, BD, Spars, MD]). The bacteria were suspended in a sterile phosphate-buffered saline (PBS). Bacteria abundance in the PBS was monitored by recording the optical density (OD) at 590 nm.^21^ The exact doses were assessed retrospectively by viable counts on 2YT agar plates.

*Plant Samples Collection*

*Rosmarinus officinalis *L*.*,* Origanum syriacum *L.,* Thymus syriacus* Boiss.,* Salvia palaestina *Benth., *Mentha piperita *L., and* Lavandula stoechas *L. (*Lamiaceae*); *Citrus aurantium *L. and* Citrus medica *L. (*Rutaceae*);* Syzygium aromaticum* L.,* Myrtus communis* L., and* Eucalyptus camaldulensis Dehnh*. (*Myrtaceae*);* Cinnamomum zeylanicum* L. and *Laurus nobilis *L. (*Lauraceae*);* Juniperus foetidissima Wild* (*Cupressaceae*);* Pelargonium roseum *L. (*Geraniaceae*);* Scilla maritima Squill *and* Allium sativum *L. (*Liliaceae*);* Pinus halepensis* Miller. (*Pinaceae*);* Artemisia herba-alba *Asso. (*Compositae*);* Anabasis haussknechtii* Boiss. (*Chenopodiaceae*);* Crataegus aronia *L. (*Rosaceae*);* Mercurialis annua *L. (*Euphorbiaceae*);* Matthiola crassifolia Boiss.* (*Brassicaceae*);* Myristica fragrans *Houtt*. *(*Myristicaceae*);* Brassica nigra *Koch. (*Cruciferae*);* Coriandrum sativum *L. (*Apiaceae*);* Zingiber officinale *Rosc*. *(*Zingiberaceae*); and *Achillea fragrantissima *Forssk. (*Asteraceae*) samples were collected during the flowering season from different regions in Syria between March and July 2010, or purchased from local markets (table 1). The samples were cleaned from any strange plants, dust, or any other contaminants.

**Table 1:** Plants and their families, collection sites, and parts used

**Scientific name**	**Plant family**	**Collection site**	**Altitude (m)**	**Collection time**	**Extracted part**	**Extract or oil**
*Rosmarinus officinalis *L*.*	*Lamiaceae*	Latakia	300	June	Aerial parts	Oil
*Origanum syriacum *L*.*	*Lamiaceae*	Kafr Nobol-Idlib	446	July	Aerial parts	Oil
*Thymus syriacus *Boiss*.*	*Lamiaceae*	Alsoja Mountain-Damascus	840	July	Aerial parts	Oil
*Salvia palaestina *Benth.	*Lamiaceae*	Alyarmouk Valley-Konaitera	800	June	Aerial parts	Oil
*Mentha* *piperita,. *L*.*	*Lamiaceae*	Latakia	300	June	Aerial parts	Oil
*Lavandula stoechas *L*.*	*Lamiaceae*	Tartous	300	June	Aerial parts	Oil
*Citrus aurantium* L.	*Rutaceae*	Latakia	300	April	Flowers	Oil
*Citrus medica *L.	*Rutaceae*	Latakia	300	April	Flowers	Oil
*Syzygium aromaticum *L.	*Myrtaceae*	Market			Flowers	Oil
*Myrtus communis* L.	*Myrtaceae*	Latakia	300	June	Leaves	Extract
*Eucalyptus camaldulensis* Dehnh.	*Myrtaceae*	Tartous	300	June	Flowering branches	Oil
*Cinnamomum zeylanicum* L.	*Lauraceae*	Market			Barks	Oil
*Laurus nobilis* L.	*Lauraceae*	Latakia	300	July	Leaves	Extract
*Juniperus foetidissima *Wild	*Cupressaceae*	Dobaya-Damascus	800	June	Leaves	Oil
*Pelargonium roseum *L.	*Geraniaceae*	Kodsaya-Damascus	916	May	Aerial parts	Extract
*Scilla maritime* Squill.	*Liliaceae*	Tartous	300	March	Bulbs	Extract
*Allium sativum *L.	*Liliaceae*	Market			Bulbs	Oil
*Pinus halepensis *Miller*.*	*Pinaceae*	Dobaya-Damascus	900	May	Leaves	Extract
*Artemisia herba-alba* Asso.	*Compositae*	Alsoja Mountain-Damascus	840	March	Aerial parts	Extract
*Anabasis haussknechtii *Boiss*.*	*Chenopodiaceae*	Alkariatain-Homs	500	March	Aerial parts	Extract
*Crataegus aronia *L.	*Rosaceae*	Alkonaitera	1100	April	Flowering branches	Extract
*Mercurialis annua* L.	*Euphorbiaceae*	Kasab-Latakia	800	March	Aerial parts	Extract
*Matthiola crassifolia* Boiss.	*Brassicaceae*	Latakia	10	March	Aerial parts	Extract
*Myristica fragrans *Houtt.	*Myristicaceae*	Market			Fruit	Oil
*Brassica nigra *Koch.	*Cruciferae*	Market			Seeds	Oil
*Coriandrum sativum *L.	*Apiaceae*	Market			Seeds	Oil
*Zingiber officinale *Rosc.	*Zingiberaceae*	Market			Rhizome	Oil
*Achillea fragrantissima* Forssk.	*Asteraceae*	Palmyra	405	July	Aerial parts	Oil

*Essential Oil Extraction*

Essential oils from fresh, clean, weighed aerial parts, flowers, leaf fruits, barks, seeds, rhizomes, and bulbs (table 1) extracted by hydro-steam distillation using the Clevenger apparatus were collected and stored in sterile vials.^22^ Briefly, 100 to 150 g of each plant was introduced in the distillation flask (1 L), which was connected to a steam generator via a glass tube and to a condenser to retrieve the oil. This was recovered in a funnel tube. Aromatic molecules of the essential oils were released from the plant material and evaporated into hot steam. The hot steam forced the plant material to release the essential oil without burning the plant material itself. Then, steam containing the essential oil was passed through a cooling system in order to condense the steam. The steam was applied for 3 h. After settling the recovered mixture, essential oil was withdrawn. The supernatant essential oil was filtered through anhydrous Na_2_SO_4_ to dry the yielded essential oil. Afterward, the essential oil was collected in tightened vials and stored in a refrigerator. For the antimicrobial activity test, several dilutions of the oils were done using dimethyl sulfoxide (DMSO).

*Preparation of Ethanolic Extracts*

Successive solvent extraction was performed for some plants (table 1). Leaves and bulbs were washed, air dried for 7-8 days, and ground into powder before they were placed into the flask of the Soxhlet apparatus for extraction using ethanol with increasing order of polarity to extract the phytoconstituents separately at 20ºC for 3-4 h. (The ethanol used was HPLC grade obtained from Sigma-Aldrich, Germany.) Whatman No.1 filter papers were then applied to filter the extracts. After that, reduced pressure was applied to evaporate and dry the filtrates, which were stored at -20ºC in labeled, sterile, screw-capped bottles.

*Antibacterial Susceptibility Assay*

Muller-Hinton Broth (MHB, Merck) medium was used to grow the test isolates for 22 h at 37°C. Final bacterial numbers were standardized to 1×10^ 6^ CFU/ml. A total of 0.1 ml of bacterial suspension was poured on each plate, containing Muller-Hinton Agar (MHA, Merck). The lawn culture was prepared by sterile cotton swab and allowed to remain in contact for 1 min. Thereafter, a 5% concentration of each plant extracts was prepared. The sterile filter paper discs (6-mm diameter) were placed on the lawn cultures, and 24 h after incubation at 37^°^C, the inhibition zone was measured in mm.

*Antibiotics Minimum Inhibitory Concentration Determination*

In order to estimate the antibiotics susceptibility, the well broth microdilution method was used with 96-well plates (TPP, Switzerland). The antibiotics were diluted twofold in LB broth^®^ (Acumedia, Michigan, USA), and the wells were inoculated with 1×10^ 6^ CFU of bacteria (in a 0.2 ml final volume). The incubation period was 24 h at 37°C. The lowest concentration that inhibited 50% of visual growth was recorded and interpreted as the MIC_50_. The MIC testing was performed according to the recommendations of the Clinical and Laboratory Standards Institute (CLSI).^23^ The range of the concentrations assayed for each antibiotic was 0.064 to 128 μg/ml. The absorbance was determined at 590 nm (Thermo-Lab Systems Reader, Finland). All the tests were performed in triplicate and then averaged. The investigated antibiotics were Ciprofloxacin, Levofloxacin, Ofloxacin, Sparfloxacin, Ceftazidime, Ceftriaxone, and Cefotaxime. Positive control was done without adding any antibiotics.

*Plants Extracts and Oils Minimum Inhibitory Concentration Determination*

The microdilution broth susceptibility assay was used.^24^ Three replicates of the serial dilutions of each essential oil were prepared in LB broth medium in 96-well microtiter plates, using a range of concentrations for each essential oil from 0.75 to 50 µl/ml. Next, 100 μl of freshly grown bacteria, standardized until a bacterial number of 1×10^ 6^ CFU/ml in LB broth was achieved, was added to each well. Positive and negative controls were also done. The plate was incubated with shaking for 24 h at 37˚C. The lowest concentration that inhibited 50% of visual growth was recorded and interpreted as the MIC_50_.

*Statistical Analysis*

Optimal concentrations for the most effective essential oils and plant extracts were estimated by Probit Analysis (SPSS Inc. 2010; Finney, 1971). Minimum concentrations to achieve 50% inhibition of the various bacteria (MIC_50_) were considered significantly different if their 95% confidential limits did not overlap.

## Results

Table 2 demonstrates that *O. syriacum. *L., *T. syriacus*,* S. aromaticum*,* C. zeylanicum*, *L. nobilis* L.,* J. foetidissima*,* A. sativum *L., and* M. fragrans *Houtt*. *had good antibacterial activities against the Gram-negative bacteria, whereas the rest of the studied extracts were ineffective.

**Table 2:** Number of Gram-negative isolates susceptible to each plant extract

	**Number of isolates susceptible to plant extracts**			
	***E. coli *** **O157:H7**	***Y. enterocolitica*** ** O9**	***Proteus*** ** spp**	***K. pneumoniae***
*Rosmarinus officinalis *L*.*	1	2	2	2
*Origanum syriacum *L*.*	12	12	13	12
*Thymus syriacus *Boiss*.*	12	15	15	11
*Salvia palaestina *Benth.	0	0	0	0
*Mentha piperita. *L*.*	1	0	2	1
*Lavandula stoechas *L*.*	3	3	5	6
*Citrus aurantium* L.	1	0	0	0
*Citrus medica *L.	1	1	0	0
*Syzygium aromaticum *L.	9	14	13	14
*Myrtus communis* L.	0	3	2	3
*Eucalyptus camaldulensis* Dehnh.	1	2	2	2
*Cinnamomum zeylanicum* L.	14	15	15	13
*Laurus nobilis* L.	14	13	13	15
*Juniperus* * foetidissima Wild*	11	11	12	13
*Pelargonium roseum *L.	2	2	3	5
*Scilla maritime* Squill.	2	1	1	2
*Allium sativum *L.	14	15	15	15
*Pinus halepensis *Miller*.*	0	0	0	0
*Artemisia herba-alba* Asso.	0	0	0	0
*Anabasis haussknechtii *Boiss*.*	0	0	0	0
*Crataegus aronia *L.	1	0	0	0
*Mercurialis annua* L.	0	0	1	0
*Matthiola crassifolia* Boiss.	3	4	2	3
*Myristica fragrans *Houtt.	13	13	13	12
*Brassica nigra *Koch.	0	0	0	0
*Coriandrum sativum *L.	3	3	2	0
*Zingiber officinale *Rosc.	3	3	4	5
*Achillea fragrantissima* Forssk.	0	0	0	0

The MIC_50_ values for these plant extracts and oils were 12.5, 12.5, 25, 12.5, 12.5, 25, 12.5, and 6.25 µl/ml, respectively, against *E. coli* O157:H7; and 1.5, 6.25, 6.25, 6.25, 6.25, 25, 6.25, and 12.5 µl/ml, respectively, against *Y.* *enterocolitica* O9; and 1.5, 3.125, 1.5, 1.5, 3.125, 12.5, 3.125, and 12.5 µl/ml, respectively, against *Proteus *spp.; and 6.25, 3.125, 1.5, 3.125, 6.25, 12.5, 6.25, and 6.25 µl/ml, respectively, against *K.* *pneumoniae* (table 3).

**Table 3:** Minimum inhibitory concentrations (MICs) for the selected essential oils and extracts against some Gram-negative bacteria

	** MIC_50_ and MIC_90_ of plant extracts (µl/ml) **							
	***E. coli *** **O157:H7**		***Y. enterocolitica *** **O9**		***Proteus*** ** spp**		***K. pneumoniae***	
	** MIC_50_**	** MIC_90_**	** MIC_50_**	** MIC_90_**	** MIC_50_**	** MIC_90_**	** MIC_50_**	** MIC_90_**
*Origanum* * syriacum.*	12.5	NA	1.5	12.5	1.5	12.5	6.25	NA
*Thymus syriacus. Boiss.*	12.5	NA	6.25	25	3.125	25	3.125	NA
*Syzygium* * aromaticum*	25	50	6.25	25	1.5	50	1.5	25
*Cinnamomum* * zeylanicum*	12.5	25	6.25	25	1.5	25	3.125	NA
*Laurus nobilis *L*.*	12.5	NA	6.25	50	3.125	50	6.25	12.5
*Juniperus* * foetidissima Wild*	25	50	25	50	12.5	50	12.5	25
*Allium sativum *L.	12.5	25	6.25	50	3.125	50	6.25	50
*Myristica* * fragrans Houtt*	6.25	50	12.5	50	12.5	NA	6.25	NA

In contrast, when studying the optimal concentrations that could inhibit 50% of the bacterial isolates, the X^ 2^ values were not significant (P>0.05) for all the studied concentrations, indicating adequate fit of the Probit regression models (table 4).

**Table 4:** Optimal inhibitory concentrations of the selected essential oils and extracts against some Gram-negative bacteria

**Bacteria**	**Plant**	** MIC_50_** ** (** **µl/ml)**	** X^ 2 ^**	***Significance***
*E. coli*	*O. syriacum.* L*.*	9.48	1.33	0.932
	*T. syriacus. Boiss.*	7.85	2.42	0.788
	*S. aromaticum*	16.11	2.8	0.732
	*C. zeylanicum*	9.48	1.33	0.932
	*L .nobilis *L*.*	20.43	6.32	0.276
	*J. foetidissima Wild*	21.82	2.98	0.703
	*A. sativum *L	8.41	1.71	0.888
	*M. fragrans Houtt.*	7.91	3.01	0.699
*Y. enterocolitica*	*O. syriacum.* L*.*	1.59	1.36	0.929
	*T. syriacus. Boiss.*	5.76	0.69	0.983
	*S. aromaticum*	5.22	1.28	0.937
	*C. zeylanicum*	5.76	0.69	0.983
	*L .nobilis *L*.*	4.30	2.99	0.702
	*J. foetidissima Wild*	12.57	1.87	0.867
	*A. sativum *L	5.52	2.05	0.842
	*M. fragrans Houtt.*	7.00	0.63	0.986
*Proteus spp.*	*O. syriacum.* L*.*	1.12	1.93	0.859
	*T. syriacus. Boiss.*	4.68	3.72	0.591
	*S. aromaticum*	2.21	4.92	0.426
	*C. zeylanicum*	1.35	1.73	0.885
	*L .nobilis *L*.*	4.68	3.72	0.591
	*J. foetidissima Wild*	6.98	0.78	0.978
	*A. sativum *L.	4.68	3.72	0.591
	*M. fragrans Houtt.*	6.03	0.63	0.986
*K. pneumoniae*	*O. syriacum.* L*.*	5.20	1.38)	0.927
	*T. syriacus. Boiss.*	3.03	3.58	0.612
	*S. aromaticum*	1.33	1.79	0.877
	*C. zeylanicum*	2.97	4.91	0.427
	*L .nobilis *L*.*	3.51	1.20	0.954
	*J. foetidissima Wild*	9.81	5.22	0.390
	*A. sativum *L.	8.75	3.86	0.570
	*M. fragrans Houtt.*	12.4	6.53	0.258

Table 5 also shows that Ceftazidime, Cefotaxime, and Ciprofloxacin were the most effective antibiotics against *E. coli *O157:H7 (MIC_50_= 0.25, 0.5, and 2 µg/ml, respectively). Moreover, Ceftazidime and Ciprofloxacin were the most effective antibiotics against *Y. enterocolitica* O9 (MIC_50_= 0.25 and 0.5 µg/ml, respectively) and against *Proteus* spp. (MIC_50_= 4 and 2 µg/ml, respectively) and Ceftriaxone, Cefotaxime, and Ciprofloxacin were the most effective antibiotics against *K. pneumoniae* (MIC_50_= 0.25, 0.25, and 0.5 µg/ml, respectively).

**Table 5:** Minimum inhibitory concentrations (MICs) of some antibiotics against Gram-negative bacteria

	** MIC_50_ and MIC_90 _of some antibiotics (µg/ml) **							
	***E. coli *** **O157:H7**		***Y. enterocolitica *** **O9**		***Proteus*** ** spp**		***K. pneumoniae***	
	** MIC_50_**	** MIC_90_**	** MIC_50_**	** MIC_90_**	** MIC_50_**	** MIC_90_**	** MIC_50_**	** MIC_90_**
Ciprofloxacin	2	64	0.5	NA	2	NA	0.5	NA
Levofloxacin	4	NA	4	NA	4	NA	4	NA
Ofloxacin	4	NA	2	64	2	NA	4	NA
Sparfloxacin	NA	NA	32	NA	32	NA	32	NA
Ceftazidime	0.25	NA	0.25	64	4	NA	4	NA
Ceftriaxone	32	NA	32	NA	32	NA	0.25	NA
Cefotaxime	0.5	NA	8	NA	64	NA	0.25	NA

## Discussion

Because of their safety and low cost as well as their impact on a large number of microbes,^25^medicinal plants may have the ability to treat bacterial resistance to many types of antibiotics. The antimicrobial effects of aromatic oils extracted from a large number of plants have been evaluated and reviewed,^26^^,^^27^ and the mechanisms that enable the natural ingredients of herbs and spices to resist microbes have been discussed.^28^ The results show that these mechanisms vary greatly depending on the components of the essential oil.^29^^,^^30^

In the present study, the efficacy of some plant extracts and oils was determined, quantitatively, by measuring the diameter of the inhibition zones around the discs (table 2). Only *O. syriacum. *L.,* T. syriacus *Boiss., *S. aromaticum *L.,* C. zeylanicum *L., *L. nobilis *L*., J. foetidissima Wild*,* A. sativum *L., and *M. fragrans *Houtt. extracts inhibited the growth of the tested bacteria. In addition, *O. syriacum. *L.,* T. syriacus* Boiss.,* S. aromaticum *L., and* C. zeylanicum* L. essential oils were the most effective, and their MIC_50_ values varied from 1.5 µl/ml to 25 µl/ml against various kinds of bacteria. Because the values of minimum bactericidal concentration (MBC) and MIC are usually very similar,^31^ it can be logically assumed that the above-mentioned plant extracts and oils have a bactericidal effect on Gram-negative bacteria, especially against* Proteus *spp. and *K.* *pneumoniae*.

The Probit Analysis (table 4) revealed that the minimum concentrations of the essential oils that could inhibit 50% of the various bacteria were *T. syriacus Boiss*. for *E. coli *O157H7 (7.85 µl/ml), O. syriacum. L. for *Proteus* spp. and *Y. enterocolitica* (1.12 and 1.59 µl/ml, respectively), and *S. aromaticum* for *K. pneumoniae* (1.33 µl/ml).

Ooi et al.^32^ reported that *Cinnamomum verum* shows excellent activities against *E. coli* and *Proteus vulgaris*. Preuss et al.^33^ found that *origanum* essential oil proves cidal to *E. coli* and *K. pneumoniae*. In addition, Barbosa et al.^34^ found that the MIC_90_ of *Origanum vulgare* essential oil is 0.46% (v/v) against *E. coli*. López et al.^35^ found that 8-10% (v/v) concentrations of *Origanum vulgare* essential oil can completely inhibit the growth of *E. coli* and other Gram-negative bacteria. Elsewhere, Mkaddem et al.^36^ reported that *Mentha* essential oils are very active against *K. pneumoniae* bacteria, whereas they are less effective against *E. coli*. Furthermore, *Mentha longifolia* oil is thought to exhibit an antimicrobial activity against some Gram-positive bacteria such as *Streptococcus mutans *and *Staphylococcus aureus*, but without affecting *Pseudomonas aeruginosa*.^37^

Since the antibacterial effectiveness of medicinal plants varies dramatically depending on the phytochemical characteristics of plant families and subfamilies, it is not surprising to note the difference in this efficacy even when using samples taken from the same plant, but from two different regions.^38^ Our results reveal that the cephalosporins were the most effective antibiotics against almost all the studied bacteria, and only Ciprofloxacin, one of the fluoroquinolones group, was effective against these bacteria.

## Conclusion

*O. syriacum. *L.,* T. syriacus* Boiss.,* S. aromaticum *L.,* C. zeylanicum *L., *J. foetidissima Wild*,* A. sativum *L., and* M. fragrans *Houtt*. *oils and *L. nobilis* L. extract were the most effective plant extracts against the Gram-negative bacteria studied in this work. These plant extracts could be a potential source of new antibacterial agents.

Further and more specific studies, in vivo, are recommended to determine the efficacy of these essential oils in the treatment of gram-negative bacterial infections. 
